# Comparison of efficacy and safety of systemic glucocorticoids and Nefecon in IgA nephropathy patients: a real-world retrospective cohort study

**DOI:** 10.3389/fimmu.2026.1865905

**Published:** 2026-09-10

**Authors:** Chenjie Gu, Jingying Zhou, Le Kang, Ming Fang

**Affiliations:** 1 Department of Nephrology, The First Affiliated Hospital of Dalian Medical University, Renal Translational Medicine Center of Liaoning Province, Dalian, Liaoning, China; 2 Medical College of Dalian University, Dalian, Liaoning, China

**Keywords:** IgA nephropathy, chronic kidney disease, glucocorticoids, Nefecon, proteinuria

## Abstract

**Background:**

This study compared the effectiveness and safety profiles of systemic glucocorticoids (sGCs) and Nefecon in the treatment of patients with immunoglobulin A nephropathy (IgAN).

**Methods:**

Patients with IgAN who had an estimated glomerular filtration rate (eGFR) ≥ 15 mL/min/1.73 m² and were treated with sGCs or Nefecon were included. The primary outcome was defined as a ≥ 30% reduction in the urine protein-to-creatinine ratio (UPCR) and a combination of partial or complete remission at 12 months. Analysis of variance (ANOVA) with a mixed-effects model and t-tests was used to compare the therapeutic effectiveness and safety profiles of sGCs and Nefecon.

**Results:**

One hundred and eight patients with IgAN were included in the study, with 54 patients receiving sGCs and 54 patients receiving Nefecon. For efficacy, the mean annual change in UPCR after treatment (sGCs versus Nefecon) was −32.01% versus −50.34%, P = 0.007. The total remission rate at 12 months (sGCs versus Nefecon) was 29.63% versus 61.11%. The percentage of patients with a ≥ 30% decline in UPCR at 12 months (sGCs versus Nefecon) was 68.52% versus 90.74%. The mean 12-month change in eGFR after treatment (sGCs versus Nefecon) was 0 versus 2.28 mL/min/1.73 m², P = 0.266. The median annualized change in eGFR after treatment (sGCs versus Nefecon) was −2.31 [−7.49, 3.75] versus 1.36 [−1.23, 6.53] mL/min/1.73 m², P = 0.002. For safety, in sGCs group, there are 3 MAKEs events while no MAKEs event in Nefecon group. Eight patients experienced severe adverse events in the sGCs group, compared with only one patient in the Nefecon group.

**Conclusion:**

Our study demonstrated that Nefecon had greater efficacy than sGCs at 12 months and a more favorable safety profile in patients with IgAN, although confirmation in larger prospective studies with longer follow-up is warranted.

## Introduction

Immunoglobulin A nephropathy (IgAN) is now recognized as one of the most common forms of primary glomerulonephritis worldwide, especially in Asia. Characterized by predominant mesangial deposition of circulating immune complexes containing galactose-deficient IgA1 (Gd-IgA1), IgAN has a range of clinical manifestations varying from asymptomatic hematuria and proteinuria to rapidly progressive kidney disease (1, 2). Patients with IgAN have a high risk of a composite outcome consisting of a ≥ 50% decline in estimated glomerular filtration rate (eGFR), kidney failure, or mortality (3). Effective therapy is urgently needed to stabilize kidney function in patients with IgAN.

The pathogenesis of IgAN may be summarized by the “4-hit hypothesis”: elevated circulating Gd-IgA1 (Hit 1), production of IgG or IgA autoantibodies (Hit 2), formation of Gd-IgA1-containing circulating immune complexes (Hit 3), and mesangial deposition and glomerular injury (Hit 4) (4). The TESTING trial indicated that systemic glucocorticoids (sGCs) reduced proteinuria in patients with IgAN by antagonizing the Hit 4 pathological pathway (5). However, the long-term benefits of sGCs for kidney outcomes remain controversial (6).

In IgA nephropathy, gut-associated lymphoid tissues (GALTs), including Peyer’s patches and mesenteric lymph nodes, are thought to be the main sites where Gd-IgA1 is overproduced by follicular B cells (7, 8). Nefecon (budesonide) is a novel delayed-release formulation of budesonide that acts on GALTs and may reduce the production of Gd-IgA1 in Peyer’s patches, thereby preventing kidney damage in IgAN. Systemic adverse events are reduced because of the targeted delivery of budesonide to intestinal immune tissues. The NefIgArd trial (NCT03643965) demonstrated that 9-month treatment with Nefecon in patients with IgAN significantly reduced proteinuria and protected kidney function, with a favorable safety profile (9).

This study aimed to compare the efficacy and safety of sGCs and Nefecon in patients with IgAN receiving optimized supportive care, which was not included in previous studies.

## Materials and methods

**Figure 1:**
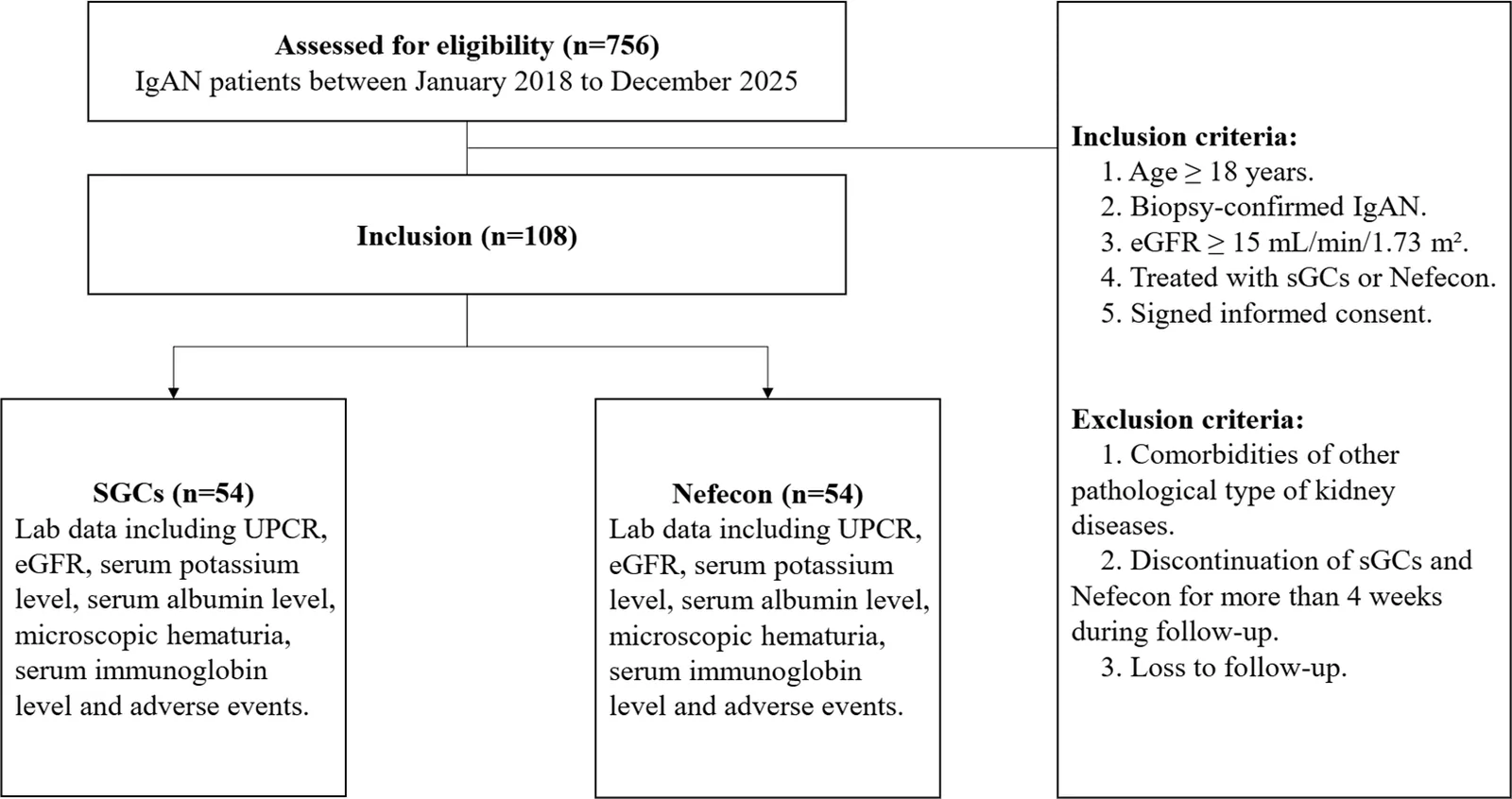
Flow chart of the study. IgAN, immunoglobulin A nephropathy; eGFR, estimated glomerular filtration rate; sGCs, systemic glucocorticoids; UPCR, urine protein-to-creatinine ratio.

This retrospective observational cohort study was conducted at the First Affiliated Hospital of Dalian Medical University between January 2018 and December 2025. All patients with IgAN were prescribed at least one type of optimized supportive care: maximally tolerated renin-angiotensin system (RAS) inhibitors, sodium-glucose cotransporter 2 (SGLT2) inhibitors, and/or a non-steroidal mineralocorticoid receptor antagonist (ns-MRA; finerenone). The inclusion criteria were as follows: age ≥ 18 years, biopsy-confirmed diagnosis of IgAN, estimated glomerular filtration rate (eGFR) ≥ 15 mL/min/1.73 m², treatment with sGCs or Nefecon with regular follow-up data, and provision of signed informed consent. Exclusion criteria included comorbidities involving other pathological types of kidney disease, discontinuation of sGCs or Nefecon for more than 4 weeks during follow-up, and loss to follow-up. Patients in the sGCs group were prescribed either oral methylprednisolone (0.6–0.8 mg/kg/day) or prednisone (0.5–1 mg/kg/day) for 6–9 months. Patients in the Nefecon group were prescribed Nefecon (16 mg/day for the first 9 months of treatment and 8 mg/day for the subsequent 3-month maintenance period). The study flow chart is shown in **Figure 1**. The primary endpoints of the study were a demonstrated ≥30% reduction in urine protein-to-creatinine ratio (UPCR), complete remission (CR), partial remission (PR), and total remission. CR was defined as UPCR < 300 mg/g in the absence of kidney function deterioration. PR was defined as UPCR < 1,000 mg/g and a reduction in urine protein of at least 50% compared with baseline, with stable kidney function. No remission (NR) was defined as not meeting the aforementioned criteria for CR or PR. Key outcomes included major adverse kidney events (MAKEs), consisting of an eGFR reduction ≥ 50%, end-stage kidney disease, and death. Severe adverse events were also observed in the study and included severe infection requiring hospitalization, new-onset diabetes, gastrointestinal hemorrhage, fracture or osteonecrosis, and cardiovascular events.

Demographic data, clinical history, medications, and laboratory results (UPCR and serum albumin and creatinine levels) were collected at baseline and follow-up visits. eGFR was calculated using the 2021 Chronic Kidney Disease Epidemiology Collaboration (CKD-EPI) equation. All patients were followed up every 2–4 months. Adverse events were also recorded. All data were collected from electronic health records.

Continuous variables with a normal distribution were expressed as the mean ± standard deviation (SD), while those with a skewed distribution were expressed as the median [interquartile range (IQR), Q1, Q3]. All data underwent normality tests to verify their distribution. UPCR values were log-transformed to achieve a normal distribution. The least squares mean (LSM) of UPCR was calculated based on the original data and expressed as the LSM (95% confidence interval [95% CI]). Analysis of variance (ANOVA) with a mixed-effects model (restricted maximum likelihood method) was used to evaluate the significance of the data and handle missing values for multiple groups. The Geisser-Greenhouse epsilon hat method was used to correct data without assuming sphericity. Independent-samples t-tests, paired-samples t-tests, and Mann-Whitney tests were used to evaluate the significance of differences between two groups. Statistical analyses and data visualizations were performed using GraphPad Prism 10.6.0 (GraphPad Software, Boston, MA, USA).

## Results

### Baseline data

Baseline characteristics of the study population are summarized in **Tables 1**, **2**. Overall, the two treatment groups were generally comparable with respect to demographic characteristics, kidney function, proteinuria, serum albumin, immunoglobulin levels, urinary microscopic red blood cell counts, pathological findings, and clinical history. Although modest differences were observed in serum potassium, sodium, and hemoglobin levels and concomitant medications, baseline disease severity was generally well balanced between the two groups.

**Table 1:** Baseline demographic, clinical, laboratory, pathological, and medical history characteristics of the study population.

Variables	sGCs (n=54)	Nefecon (n=54)	P value
Demographic data, n (%)			
Age, years	41 [34, 55]	43 [34, 51]	0.929
≥50 years	17 (31.48%)	16 (29.63%)	
35–50 years	22 (40.74%)	24 (44.44%)	
<35 years	15 (27.78%)	14 (25.93%)	
Female/Male	36 (66.67%)/18 (33.33%)	30 (55.56%)/24 (44.44%)	
Age of biopsy, years	41 [34, 55]	37 [30, 45]	0.091
Median follow-up, years	3.00 [1.00, 5.25]	1.00 (0)	< 0.001
Laboratory data			
BMI, kg/m^2^	24.45 [22.25, 28.70]	26.65 [24.13, 29.55]	0.145
SBP, mmHg	135 [120, 140]	130 [120, 150]	0.346
DBP, mmHg	85 [80, 90]	81 [80, 96]	0.902
UPCR, mg/g	1853.50 [1102.75, 3560.25]	1619.40 [1050.00, 2746.75]	0.501
≥ 1,500 mg/g	36 (66.67%)	29 (53.70%)	
< 1,500 mg/g	18 (33.33%)	25 (46.30%)	
eGFR, mL/min/1.73 m²	63.18 (18.60)	56.68 (19.57)	0.080
CKD 1	6 (11.11%)	6 (11.11%)	
CKD 2	27 (50.00%)	14 (25.93%)	
CKD 3a	13 (24.07%)	15 (27.78%)	
CKD 3b	6 (11.11%)	12 (22.22%)	
CKD 4	2 (3.70%)	7 (12.96%)	
Serum albumin, g/L	39.7 (4.3)	39.9 (5.3)	0.832
Serum creatinine, μmol/L	101 [90, 123]	125 [94, 155]	0.073
Serum potassium, mmol/L	4.03 (0.42)	4.36 (0.48)	< 0.001
Serum hemoglobin, g/L	124 (19)	134 (18)	0.006
Serum uric acid, μmol/L	397 [347, 463]	383 [327, 480]	0.196
Serum sodium, mmol/L	140 [139, 142]	142 [140, 143]	0.002
TG, mmol/L	1.78 [1.04, 2.57]	1.85 [1.48, 2.36]	0.502
LDL, mmol/L	2.81 [2.47, 3.52]	3.02 [2.40, 3.36]	0.761
HDL, mmol/L	1.11 [0.99, 1.40]	1.19 [0.95, 1.52]	0.485
TC, mmol/L	5.20 [4.69, 6.04]	5.24 [4.46, 6.16]	0.481
Urinary microscopic RBCs, cells/HP	8 [3, 21]	6 [2, 18]	0.419
Serum IgA, g/L	2.99 (0.83)	3.40 (1.20)	0.133
Serum IgM, g/L	1.13 (0.60)	1.18 (0.54)	0.737
Serum IgG, g/L	9.88 (2.98)	8.87 (2.54)	0.159
Pathology, n (%)			
Lee classification			
I	0 (0.00%)	0 (0.00%)	
II	4 (7.41%)	3 (5.56%)	
III	23 (42.59%)	16 (29.63%)	
IV	25 (46.30%)	21 (48.89%)	
V	1 (1.85%)	0 (0.00%)	
Unknown	1 (1.85%)	14 (25.93%)	
Oxford histological score			
M			
0	9 (16.67%)	2 (3.70%)	
1	32 (59.26%)	24 (44.44%)	
E			
0	39 (72.22%)	24 (44.44%)	
1	2 (3.70%)	2 (3.70%)	
S			
0	3 (5.56%)	1 (1.85%)	
1	38 (70.37%)	25 (46.30%)	
T			
0	8 (14.81%)	2 (3.70%)	
1	18 (33.33%)	11 (20.37%)	
2	15 (27.78%)	13 (24.07%)	
C			
0	22 (40.74%)	17 (31.68%)	
1	19 (35.19%)	8 (16.67%)	
2	0 (0.00%)	0 (0.00%)	
Unknown	13 (24.07%)	28 (51.85%)	
Clinical history, n (%)			
Hypertension	31 (57.41%)	35 (64.81%)	
Cardiovascular diseases	0 (0.00%)	0 (0.00%)	
Stroke	2 (3.70%)	0 (0.00%)	
Gout	13 (24.07%)	7 (12.96%)	
Diabetes	0 (0.00%)	2 (3.70%)	

Data are presented as mean (standard deviation), median [interquartile range], or number (percentage), as appropriate. Estimated glomerular filtration rate (eGFR) was calculated using the 2021 CKD-EPI equation. IgAN, immunoglobulin A nephropathy; sGCs, systemic glucocorticoids; BMI, body mass index; SBP, systolic blood pressure; DBP, diastolic blood pressure; UPCR, urine protein-to-creatinine ratio; eGFR, estimated glomerular filtration rate; CKD, chronic kidney disease; TG, triglyceride; LDL, low-density lipoprotein; HDL, high-density lipoprotein; TC, total cholesterol; RBCs, red blood cells; IgA, immunoglobulin A; IgM, immunoglobulin M; IgG, immunoglobulin G.

**Table 2:** Baseline concomitant medications and immunosuppressive therapies in the two treatment groups.

Variables	sGCs (n=54)	Nefecon (n=54)	P value
Concomitant medication, n (%)			
RAS inhibitors	54 (100.00%)	47 (87.03%)	
ARBs	51 (94.44%)	33 (70.21%)	
ACE inhibitors	3 (5.56%)	1 (2.13%)	
ARNI (sacubitril-valsartan)	0 (0.00%)	13 (27.66%)	
SGLT2 inhibitors	7 (12.96%)	44 (81.48%)	
ns-MRA (finerenone)	0 (0.00%)	29 (53.70%)	
α-blockers	0 (0.00%)	0 (0.00%)	
β-blockers	8 (14.81%)	3 (5.56%)	
CCBs	22 (40.74%)	11 (20.37%)	
Diuretics	5 (9.26%)	7 (12.96%)	
Statins	21 (38.89%)	11 (20.37%)	
Ezetimibe	0 (0.00%)	4 (7.40%)	
Antiplatelet drugs	2 (3.57%)	0 (0.00%)	
Febuxostat	13 (24.07%)	7 (12.96%)	
Current glucocorticoids	54 (100.00%)	0 (0.00%)	
Methylprednisolone	17 (31.48%)	0 (0.00%)	
Prednisone	37 (68.52%)	0 (0.00%)	
Current Nefecon	0 (0.00%)	54 (100.00%)	
Current immunosuppressants			
Hydroxychloroquine	4 (7.41%)	0 (0.00%)	
Tacrolimus	1 (1.85%)	0 (0.00%)	
Telitacicept	0 (0.00%)	1 (1.85%)	
Leflunomide	2 (3.70%)	0 (0.00%)	
Cyclophosphamide	1 (1.85%)	0 (0.00%)	
Mycophenolate mofetil	17 (31.48%)	2 (3.70%)	
Previous glucocorticoids	0 (0.00%)	20 (37.03%)	
Previous immunosuppressants			
Hydroxychloroquine	0 (0.00%)	0 (0.00%)	
Tacrolimus	0 (0.00%)	0 (0.00%)	
Telitacicept	0 (0.00%)	2 (3.70%)	
Leflunomide	0 (0.00%)	0 (0.00%)	
Cyclophosphamide	0 (0.00%)	2 (3.70%)	
Mycophenolate mofetil	0 (0.00%)	3 (5.56%)	

RAS, renin–angiotensin system; ARB, angiotensin II receptor blocker; ACEI, angiotensin-converting enzyme inhibitor; ARNI, angiotensin receptor–neprilysin inhibitor; SGLT2, sodium-glucose cotransporter 2; ns-MRA, non-steroidal mineralocorticoid receptor antagonist; CCB, calcium channel blocker. Data are presented as number (percentage).

### Efficacy outcomes

**Figure 2:**
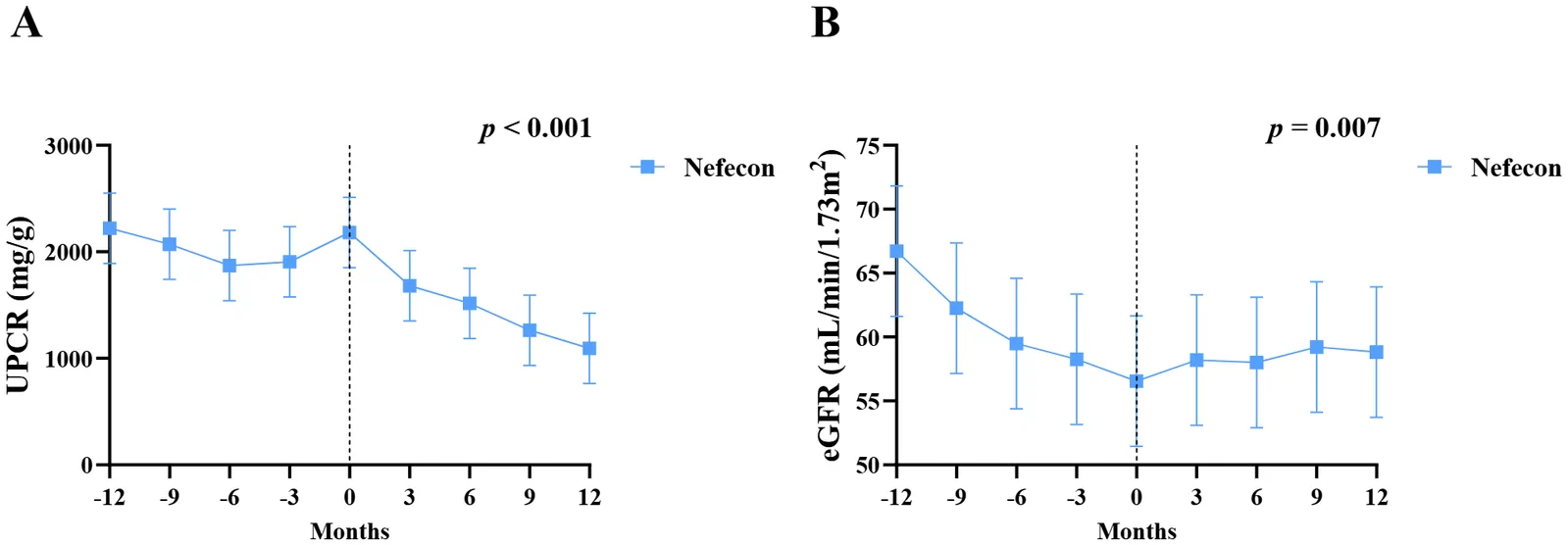
Changes in UPCR and eGFR in the Nefecon group from 12 months before baseline to 12 months after baseline. **(A)** LSM of UPCR. **(B)** LSM of eGFR. Points represent means; bars represent 95% confidence intervals. UPCR, urine protein-to-creatinine ratio. eGFR, estimated glomerular filtration rate. LSM, least squares mean.

The longitudinal efficacy data for the Nefecon group from 12 months before baseline to 12 months after baseline are shown in **Figures 2A, B**. The LSM of UPCR was 2,221.54 mg/g at 12 months before baseline, 2,180.95 mg/g at baseline, and 1,082.99 mg/g at 12 months after baseline. The annual change in UPCR was −1.83% before treatment and −50.34% after treatment (P < 0.001). The LSM of eGFR was 66.71 mL/min/1.73 m² at 12 months before baseline, 56.55 mL/min/1.73 m² at baseline, and 58.83 mL/min/1.73 m² at 12 months after baseline. The annual change in eGFR was −10.16 mL/min/1.73 m² before treatment and 2.28 mL/min/1.73 m² after treatment (P = 0.007).

**Figure 3:**
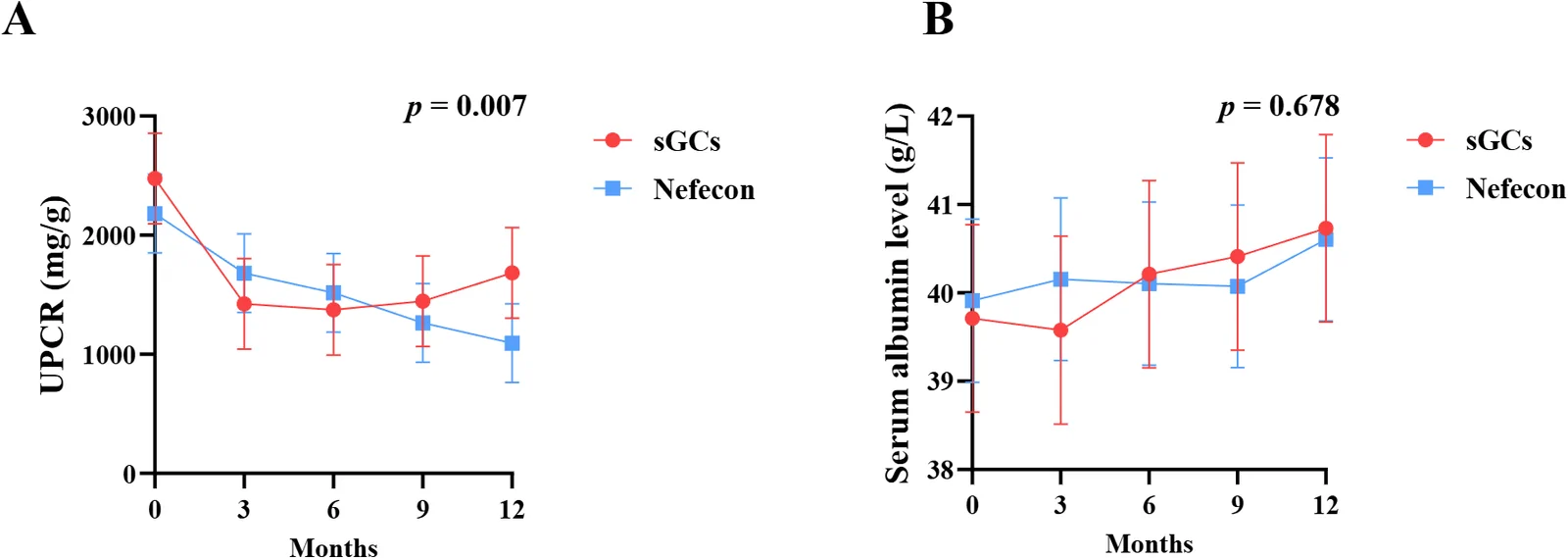
Changes in UPCR and serum albumin levels from baseline to 12 months after baseline. **(A)** LSM of UPCR. **(B)** LSM of serum albumin level. Points represent means; bars represent 95% confidence intervals. UPCR, urine protein-to-creatinine ratio; eGFR, estimated glomerular filtration rate; LSM, least squares mean; sGCs, systemic glucocorticoids.

The comparative efficacy data for UPCR and serum albumin levels in the sGCs and Nefecon groups from baseline to 12 months after baseline are shown in **Figures 3A, B**. The LSM of UPCR at baseline (sGCs versus Nefecon) was 2,475.08 versus 2,180.95 mg/g. At 12 months, the LSM of UPCR was 1,682.83 versus 1,082.99 mg/g. The mean annual change after treatment (sGCs versus Nefecon) was −32.01% versus −50.34% (P = 0.007). The LSM of serum albumin at baseline (sGCs versus Nefecon) was 39.7 versus 39.9 g/L, and at 12 months, it was 40.7 versus 40.6 g/L. The mean annual change was 1.0 versus 0.7 g/L (P = 0.678).

**Figure 4:**
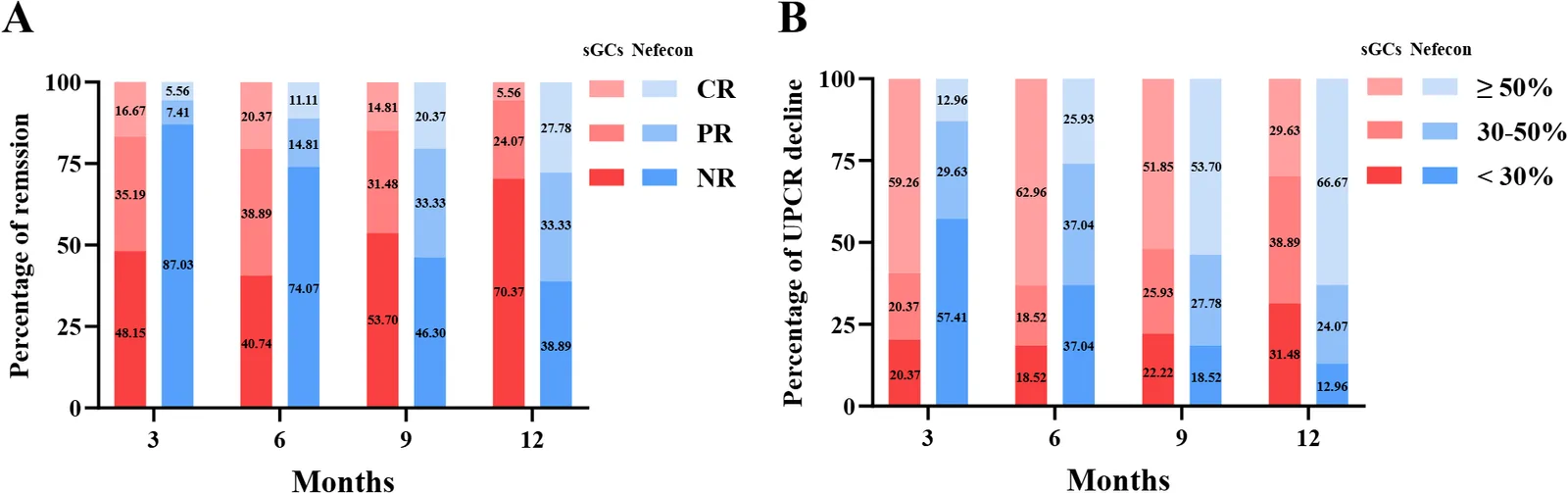
Proportions of remission and UPCR decline from baseline at different visits. **(A)** Proportion of remission. **(B)** Proportion of UPCR decline percentages. Bars represent percentages. UPCR, urine protein-to-creatinine ratio; CR, complete remission; PR, partial remission; NR, no remission; sGCs, systemic glucocorticoids.

Remission and UPCR decline profiles are shown in **Figures 4A, B**. Over the 12-month period, the Nefecon group exhibited progressively improving outcomes in both complete and partial remission compared with the sGCs group. The total remission rate at 12 months (sGCs versus Nefecon) was 29.63% versus 61.11%. Similarly, the percentage of patients with a ≥ 30% decline in UPCR at 12 months (sGCs versus Nefecon) was 68.52% versus 90.74%.

**Figure 5:**
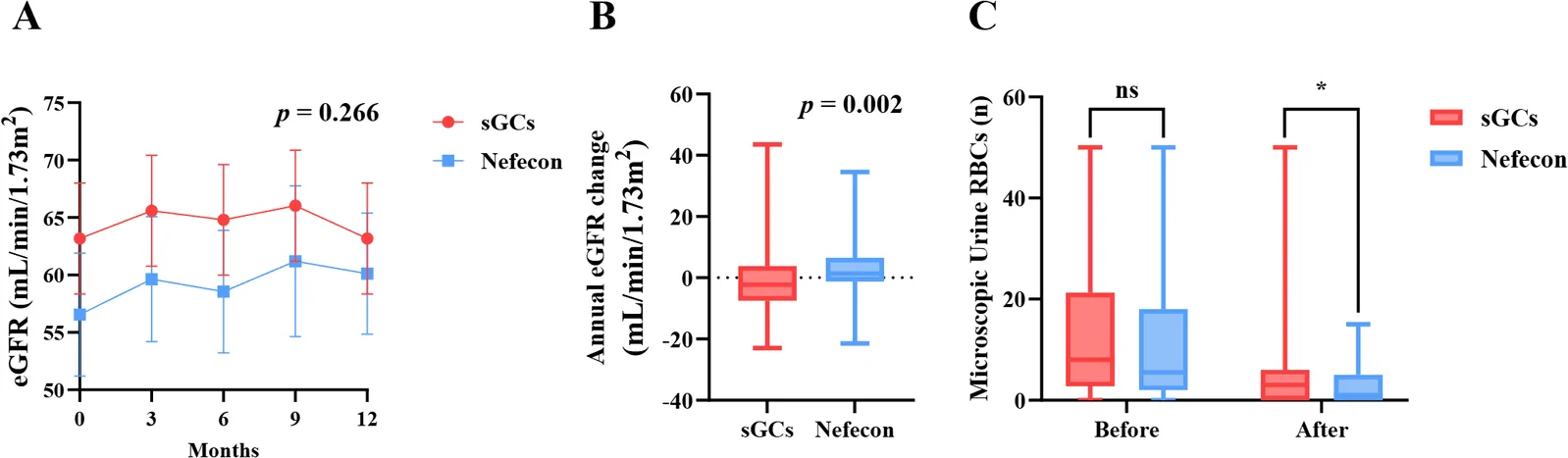
Mean change in eGFR, annual change in eGFR (each patient’s last recorded eGFR value annualized by the patient’s total follow-up years since administration of sGCs or Nefecon), and microscopic RBC counts before and after 12 months of treatment. **(A)** LSM of eGFR. **(B)** Annualized eGFR change. **(C)** Microscopic RBC counts. Points represent means. Error bars represent standard deviations for **(A)**. Boxes represent medians and interquartile ranges. Error bars represent minimum and maximum values for **(B, C)**. eGFR, estimated glomerular filtration rate; LSM, least squares mean; RBCs, red blood cells; ns, not significant. *P < 0.05.

The comparative efficacy data for eGFR, annual change in eGFR, and microscopic RBC counts before and after 12 months of treatment are shown in **Figures 5A–C**. The LSM of eGFR at baseline (sGCs versus Nefecon) was 63.18 versus 56.55 mL/min/1.73 m², and at 12 months, it was 63.18 versus 58.83 mL/min/1.73 m² (P = 0.266). The median annualized change in eGFR after treatment (sGCs versus Nefecon) was −2.31 [−7.49, 3.75] versus 1.36 [−1.23, 6.53] mL/min/1.73 m² (P = 0.002). The median microscopic RBC count at baseline (sGCs versus Nefecon) was 8 [3, 21] versus 6 [2, 18] cells/HP (P = 0.419), and at 12 months, it was 3 [0, 6] versus 1 [0, 5] cells/HP (P = 0.022).

### Safety outcomes

Safety outcomes are summarized in **Table 3**. The incidence of major adverse kidney events (MAKEs) was low during the 12-month follow-up. Three patients (5.56%) in the sGCs group experienced an eGFR decline of ≥ 50%, whereas no MAKEs occurred in the Nefecon group. No severe adverse events leading to treatment discontinuation or death were reported during the study period. Eight patients (14.81%) in the sGCs group experienced severe adverse events, including six cases of severe infection, one case of gastrointestinal hemorrhage, and one case of fracture or osteonecrosis. In contrast, only one patient (1.85%) in the Nefecon group developed a severe adverse event (new-onset diabetes).

**Table 3:** Observed severe adverse events in 108 patients with IgAN.

Variables	sGCs (n=54)	Nefecon (n=54)
Severe adverse events	8 (14.81%)	1 (1.85%)
Severe infection	6 (11.11%)	0 (0.00%)
New-onset diabetes	0 (0.00%)	1 (1.85%)
Gastrointestinal hemorrhage	1 (1.85%)	0 (0.00%)
Fracture or osteonecrosis	1 (1.85%)	0 (0.00%)
Cardiovascular events	0 (0.00%)	0 (0.00%)

IgAN, immunoglobulin A nephropathy; sGCs, systemic glucocorticoids. Data are recorded as number (percentage).

**Figure 6:**
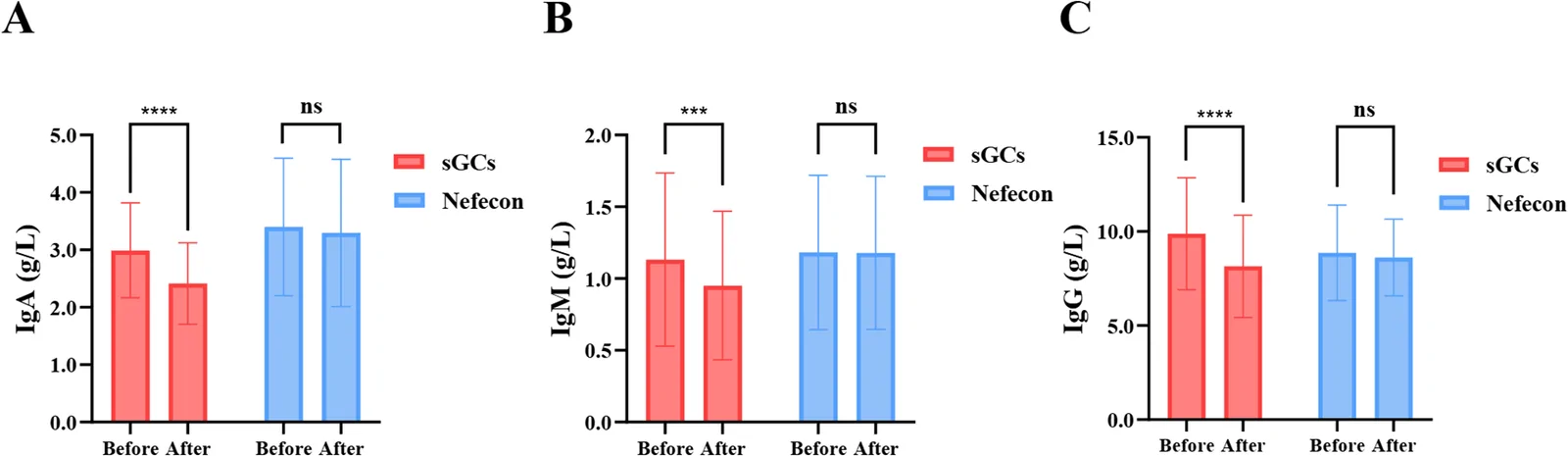
Mean serum IgA, IgM, and IgG levels before and after 12 months of treatment. **(A)** Mean serum IgA levels. **(B)** Mean serum IgM levels. **(C)** Mean serum IgG levels. Points represent means; error bars represent standard deviations. sGCs, systemic glucocorticoids; IgA, immunoglobulin A; IgM, immunoglobulin M; IgG, immunoglobulin G; ns, not significant. ***P < 0.001; ****P < 0.0001.

The comparative safety data for serum immunoglobulin A (IgA), serum immunoglobulin M (IgM), and serum immunoglobulin G (IgG) from baseline to 12 months after baseline are shown in **Figures 6A–C**. The mean serum IgA level at baseline (sGCs versus Nefecon) was 2.99 (0.83) versus 3.40 (1.20) g/L, and at 12 months, it was 2.42 (0.71) versus 3.30 (1.28) g/L. The 12-month change after treatment was significant in the sGCs group (P < 0.0001) but not in the Nefecon group (P = 0.383). The mean serum IgM level at baseline (sGCs versus Nefecon) was 1.13 (0.60) versus 1.18 (0.54) g/L, and at 12 months, it was 0.95 (0.52) versus 1.18 (0.53) g/L. The change in IgM was significant in the sGCs group (P = 0.0003) but not in the Nefecon group (P = 0.963). The mean serum IgG level at baseline (sGCs versus Nefecon) was 9.88 (2.98) versus 8.87 (2.54) g/L, and at 12 months, it was 8.16 (2.72) versus 8.62 (2.03) g/L. The change in IgG was also significant in the sGCs group (P < 0.0001) but not in the Nefecon group (P = 0.544).

## Discussion

IgAN is an autoimmune disease in which circulating immune complexes play a significant pathogenic role. Nefecon, a novel targeted-release formulation of budesonide, was recommended in the 2025 Kidney Disease: Improving Global Outcomes (KDIGO) clinical practice guideline for IgAN based on its established efficacy and safety profile (10, 11). Compared with sGCs, Nefecon has demonstrated a greater long-term reduction in proteinuria and better preservation of kidney function. This is likely attributable to its mechanism of action, which involves reducing circulatory Gd-IgA1 levels, decreasing the production of IgG autoantibodies, and inhibiting the formation of Gd-IgA1-containing circulating immune complexes (Hits 1, 2, and 3). Previous research suggests that early changes in Gd-IgA1 or poly-IgA, especially poly-IgA, were associated with future reductions in proteinuria, supporting the potential of Gd-IgA1 and poly-IgA as biomarkers for predicting the response to Nefecon in IgAN (12). However, existing published data on Nefecon consist primarily of small-sample studies or case reports, with no direct comparisons with sGCs in a real-world setting. Furthermore, considering the overlapping pathophysiological mechanisms between IgAN and IgA vasculitis (IgAV), strategies targeting the reduction of circulating galactose-deficient IgA1 are also of significant clinical interest in adult IgAV. Systemic glucocorticoids conventionally remain the first-line therapy for adult IgAV, but targeted therapies such as Nefecon may offer new approaches to improve clinical outcomes and minimize glucocorticoid-related toxicity in this related condition (13).

To address this gap, we conducted a retrospective analysis comparing the effectiveness and safety of these two treatment regimens in patients with primary IgAN. Our findings indicate that patients treated with Nefecon were more likely to achieve a reduction in proteinuria. At the 12-month endpoint, the Nefecon group had significantly higher rates of both partial and complete remission compared with the sGCs group. Both treatments were well tolerated, with a comparable incidence of adverse events. Therefore, Nefecon represents a promising alternative treatment option for primary IgAN. Further studies with extended follow-up durations are warranted to confirm these observations and assess long-term outcomes. Clinical trial data for Nefecon have demonstrated a promising efficacy profile, with a predictable pattern of adverse events. Treatment with Nefecon for 9 months substantially reduced proteinuria in Part A of the Phase 3 trial and in the Phase 2b trial. A near-complete prevention of deterioration in renal function was observed at 12 months in patients at greatest risk of rapid disease progression, which was consistent with our results (14, 15). A recently published real-world cohort study also demonstrated that Nefecon may rapidly reduce proteinuria and preserve eGFR in routine clinical practice, corroborating its broad applicability and efficacy outside tightly controlled randomized trial settings (16).

In our cohort, all patients maintained on the 16 mg dose of Nefecon during the 9-month treatment period and the 8 mg dose during the 3-month maintenance period exhibited sustained clinical benefits. As a highly specific targeted-release agent, Nefecon is hypothesized to provide durable suppression of autoantibody production and more effective remission induction in IgAN through a reduction in pathogenic galactose-deficient IgA1 production (17). A previous study suggested that the 2-year reduction in total eGFR slope with Nefecon was estimated to be 62% compared with supportive care alone (18). This hypothesis awaits confirmation in future prospective studies with prolonged follow-up.

Regarding safety, a recent comprehensive review corroborated that targeted-release budesonide minimizes systemic glucocorticoid exposure, thereby offering a highly favorable safety profile without significantly suppressing systemic immunoglobulins (19). In our study, we showed that Nefecon was well tolerated with few serious adverse events (AEs). Only one patient developed new-onset diabetes. Nefecon demonstrated a favorable safety profile, with no severe clinical events identified. Stable serum immunoglobulin levels were also observed in the Nefecon group. The identification of AEs and high-risk events offers important insights for early clinical intervention in high-risk IgAN (20). In brief, together with the published studies, our findings suggest that Nefecon is safe for treating IgAN.

Beyond traditional comparative effectiveness research, the integration of real-world clinical data with machine learning approaches may provide new opportunities for individualized risk assessment in nephrology. Recent studies have demonstrated that routinely collected clinical variables can be used to develop predictive models for kidney-related adverse events and support proactive clinical management (21, 22). Such approaches may be valuable in future IgAN research for integrating baseline characteristics, biomarkers, and treatment responses to identify patients most likely to benefit from targeted therapies.

Several limitations of our study should be acknowledged. First, this was a retrospective study with a small sample size and a limited follow-up period. Although our findings suggest favorable effects of Nefecon on proteinuria remission and preservation of renal function, the limited number of major adverse kidney events (MAKEs) reduced the statistical power to detect differences in hard renal endpoints between treatment groups. Therefore, the observed renoprotective effects should be interpreted with caution and considered hypothesis-generating rather than definitive evidence. Although baseline clinical characteristics, including renal function and proteinuria levels, were generally comparable between the groups, treatment allocation was not randomized. Therefore, differences in concomitant medications, particularly the higher use of SGLT2 inhibitors and non-steroidal mineralocorticoid receptor antagonists in the Nefecon group, may have introduced residual confounding. The independent treatment effect of Nefecon should therefore be interpreted with caution. Further research should use a prospective, randomized design with an extended follow-up period. In addition, patients in China who did not have access to Nefecon because of stock shortages were allowed to reduce the dose from 16 mg/day to 8 mg/day for 1–2 months during the first 9 months of treatment. Finally, we did not compare dynamic changes in serum Gd-IgA1 and other IgAN biomarkers during different treatment periods; these changes should be further examined in future studies.

In conclusion, our study demonstrated that Nefecon had greater efficacy than sGCs at 12 months and a more favorable safety profile in patients with IgAN, although confirmation in larger prospective studies with longer follow-up is warranted.

## Data Availability

The original contributions presented in the study are included in the article/Supplementary Material. Further inquiries can be directed to the corresponding authors.
